# Phytoplasmas: Molecular Characterization and Host–Pathogen Interactions

**DOI:** 10.3390/biology13090735

**Published:** 2024-09-20

**Authors:** Wei Wei, Yan Zhao, Fabio Quaglino

**Affiliations:** 1 Molecular Plant Pathology Laboratory, Beltsville Agricultural Research Center, Agricultural Research Service, United States Department of Agriculture, Beltsville, MD 20705, USA; yan.zhao@usda.gov; 2 Department of Agricultural and Environmental Sciences–Production, Landscape, Agroenergy, University of Milan, 20122 Milan, Italy; fabio.quaglino@unimi.it

Phytoplasmas are small, wall-less bacteria that infect many plant species and multiply within phloem-feeding insects of the Hemiptera order. The transkingdom life cycle of phytoplasmas contributes to the complexity of their pathosystem and epidemiology, spanning diverse ecological environments. This Special Issue, titled “Phytoplasmas: Molecular Characterization and Host–Pathogen Interactions”, compiles ten articles, including nine original research papers and one review. These articles offer intriguing perspectives and findings that will stimulate new approaches in both ongoing and fresh research in this dynamic field.

## 1. Genetic Diversity, Genomics, Physiology, and Epidemiology of Grapevine-Associated Phytoplasmas

In a detailed genomic study, Debonneville et al. successfully assembled the complete circular genome of the *Flavescence Dorée* (FD) phytoplasma, a major pathogen affecting grapevines across Europe [1]. The genome, consisting of 654,223 base pairs, revealed a significantly low level of genome plasticity. In contrast to the genomes of most other phytoplasmas, this FD phytoplasma genome is characterized by the absence of potential mobile units (PMUs) and a minimal number of phage-derived segments. Such genomic stability may indicate that FD phytoplasma is a highly specialized pathogen adapted to a narrow ecological niche with specific interactions with its grapevine hosts and the insect vector *Scaphoideustitanus* [1].

Rigamonti et al. conducted an epidemiological investigation in northwestern Italy, focusing on the spread of FD phytoplasmas [2]. This study identified the Map-M54 genotype as the sole phytoplasma strain affecting grapevines in the region. This study also uncovered several new potential reservoir plants that harbor different FD phytoplasma strains, indicating that the disease is not confined to vineyards but involves a broader ecosystem, including wild plants and various insect vectors. These findings broaden the understanding of FD’s epidemiology, emphasizing the complexity of its spread and the need for integrated management strategies [2]. The findings presented by Debonneville et al. [1] and Rigamonti et al. [2] highlight the genetic and ecological diversity within FD phytoplasma populations, with some strains exhibiting specific host–pathogen interactions, while others operate within a broader environmental network. Effective control of FD requires addressing both vineyard and non-vineyard plant hosts to mitigate the risk of disease transmission and protect grapevine health [2].

A study on the impact of FD phytoplasma on grapevine physiology conducted by Rizzoli et al. uncovered significant reductions in xylem growth, particularly during drought years, in infected vines [3]. This study revealed that such affected grapevines often fail to produce complete xylem rings, a critical water and nutrient transport component. Additionally, severe disruptions in phloem tissue structure were observed, including disarrangement and incomplete xylem formation in symptomatic vines. These observations suggest that FD infection not only impairs normal growth processes but also exacerbates the effects of environmental stressors like drought on grapevines [3]. This research highlights the need for proactive and effective management strategies that address both the direct effects of phytoplasma and its interactions with environmental factors to protect vineyard health and productivity.

Jamshidi et al. carried out a study on multilocus genotyping of ‘*Candidatus* Phytoplasma solani’ associated with grapevine bois noir (BN) in Iranian vineyards [4]. This research offers critical insights into the genetic diversity and spread of the pathogen. By analyzing samples from various vineyards, this study identified six known vmp1 variants and discovered two new ones, V24 and V27, with the tuf-b/V24/stamp III genotype emerging as the most prevalent, mainly linked to the weed *Convolvulus arvensis*. This finding underlines the significant role of specific weeds as reservoirs, contributing to the spread of the disease in vineyards. The study also revealed complex three-way interactions among the phytoplasma, its host plants, and insect vectors, highlighting the need for integrated management strategies targeting vectors and reservoir plants. These findings are helpful for developing more effective control measures to protect vineyards in regions where BN poses a severe threat to viticulture [4].

## 2. Phytoplasmas Affect Other Crops

Citrus: In Hainan, China, a study conducted by Yu et al. identified the presence of two subgroups of phytoplasmas (16SrII-V and 16SrXXXII-D) and ‘*Candidatus* Liberibacter asiaticus’ in *Citrus maxima*, a key citrus crop [5]. This study marks the first instance of detecting mixed infections involving 16SrII-V phytoplasmas and ‘*Candidatus* Liberibacter asiaticus’ in *Citrus maxima* in China, with the affected plants accounting for 7.4% of the samples analyzed. The discovery of these mixed infections sheds light on the intricate interactions between multiple pathogens within citrus plants, emphasizing the complexity of disease dynamics in these economically significant crops. These findings have substantial implications for the citrus industry, particularly in regions like Hainan, where understanding and addressing the co-occurrence of multiple pathogens can lead to more effective and targeted strategies for disease monitoring, prevention, and control, ultimately helping to mitigate economic losses and support sustainable agricultural practices [5].

Sesame: Verma et al. focused on the epigenetic effects of phytoplasma infection in sesame plants and conducted a comprehensive DNA methylomic analysis using whole-genome bisulfite sequencing (WGBS) [6]. The study revealed significant changes in DNA methylation patterns, particularly in plants exhibiting phyllody symptoms, which showed global cytosine hypomethylation, especially in the CHH context. In contrast, sesame plants with little leaf symptoms exhibited DNA methylation levels comparable to healthy plants. The study also identified differentially methylated regions in intergenic, promoter, exonic, and intronic regions. The differential methylations primarily occurred in genes involved in development and defense processes. This research enhances our understanding of the epigenetic mechanisms underlying phytoplasma-induced symptoms and offers potential avenues for breeding disease-resistant sesame varieties, thereby improving crop resilience and productivity.

Sandalwood spike: The study on sandalwood spike disease (SSD) conducted by Kirdat et al. provided new insights into the transmission pathways of this devastating phytoplasma disease [7]. By utilizing real-time PCR, researchers demonstrated that SSD phytoplasma can be transmitted vertically through seeds, with a significant percentage of seedlings grown from infected seeds testing positive for the pathogen. This finding is alarming as it suggests the potential for the disease to spread beyond current areas through the commercial distribution of infected seeds and seedlings, further threatening sandalwood populations. However, since phytoplasma seed transmission remains an unsettled area of research, we recommend exercising caution until these results are confirmed by at least two independent laboratories to ensure definitive evidence.

## 3. Advances in Phytoplasma Detection and Taxonomy

In a comparative study of traditional and next-generation sequencing (NGS) methods for phytoplasma detection, Trivellone et al. demonstrated that anchored hybrid enrichment (AHE) outperforms PCR-based approaches in detecting and characterizing phytoplasmas [8]. By analyzing DNA from leafhoppers collected worldwide, the study identified three new phytoplasma subgroups and three potential new groups that traditional methods had missed. This highlights the vast, previously unrecognized diversity of phytoplasmas in natural ecosystems and emphasizes the importance of modern molecular techniques for uncovering new phytoplasma threats and their insect vectors. These findings are crucial for advancing our understanding of phytoplasma diversity and disease epidemiology.

A review of phytoplasma taxonomy by Wei and Zhao [9] highlighted the significant advancements in nomenclature, classification, and identification of phytoplasmas. The updated 2022 phytoplasma taxonomy guidelines now incorporate whole genome average nucleotide identity (ANI) and a refined 16S rRNA gene identity threshold, enhancing the precision of naming and classifying ‘*Candidatus* Phytoplasma’ species. These developments are crucial to the more accurate identification of phytoplasmas, which is essential for diagnosing and managing plant diseases caused by these pathogens. The improved taxonomy also facilitates global research collaboration and helps develop targeted strategies for controlling phytoplasma-associated diseases, ultimately reducing their negative economic and environmental impacts.

Trivellone et al. reported a proactive approach using the DAMA (document, assess, monitor, act) protocol to anticipate and manage the risk of phytoplasma disease outbreaks. Applied in Bavaria, Germany, the study identified potential phytoplasma threats by detecting two ‘*Candidatus* Phytoplasma asteris’-related strains in insect vectors and assessing their risk of causing disease in key crops through phylogenetic analysis. This approach emphasizes the importance of early detection and targeted monitoring to prevent outbreaks, offering a significant advancement in plant disease management that can protect agricultural productivity [10].

In conclusion, this Special Issue enhances our understanding of phytoplasma biology and host interactions, highlighting the need for ongoing research to address their impact on plant health. We hope that these studies inspire further exploration and cross-disciplinary collaboration in this evolving area of research.
